# Surgical Management of Retro-Odontoid Cystic Mass with Cervicomedullary Compression

**DOI:** 10.1155/2021/5575181

**Published:** 2021-05-20

**Authors:** Mark K. Lyons, Matthew T. Neal, Maziyar Kalani, Naresh P. Patel

**Affiliations:** Department of Neurological Surgery, Mayo Clinic Arizona, Phoenix, AZ 85054, USA

## Abstract

Retro-odontoid cysts are a rare cause of cervicomedullary compression. The etiology of these lesions is not completely understood. Previous trauma and instability at the cervicomedullary junction may be the precipitating event in the development of retro-odontoid cysts in rare cases. We discussed the neurosurgical evaluation of a patient who presented with progressive and rapid neurological deterioration secondary to cervicomedullary compression. Posterior occipitocervical fusion was performed. The patient made an excellent neurological recovery, and postoperative imaging studies demonstrated resolution of the compression and intramedullary cyst.

## 1. Introduction

Retro-odontoid cysts are rare clinical entities. Mechanisms proposed at the etiology for these lesions include chronic instability and pseudogout [1–3]. These lesions can result in significant cervicomedullary compression and resultant neurologic deficits. Various surgical approaches have been reported to address the compression and reverse the neurologic deficits [1–8]. We present the case of an adult male presenting with acute neurologic deterioration and discuss the surgical management.

## 2. Case Report

**Figure 1:**
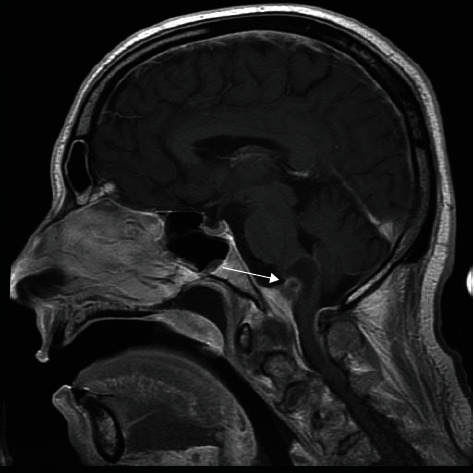
Preoperative MR brain T1-weighted sagittal image demonstrates a cystic mass arising at the C1-C2 level extending cephalad traversing the foramen magnum. The mass insinuates between the intradural segments of the vertebral arteries bilaterally causing severe compression at the cervicomedullary junction with the associated cystic area in the caudal medulla.

**Figure 2:**
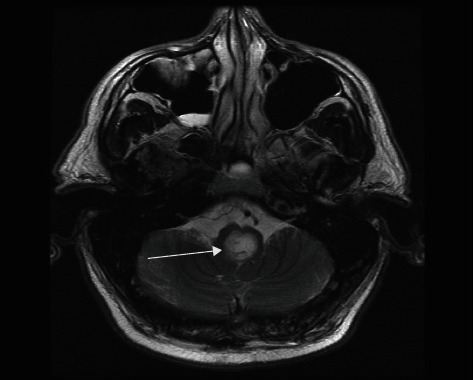
Preoperative MR brain T2-weighted axial image demonstrates a large cyst in the caudal medulla.

We report the case of a 60-year-old male with a past medical history significant for hyperlipidemia, insomnia, and anxiety. The patient developed the onset of rapidly progressive motor weakness beginning in the lower extremities and ascending over the course of several days. He presented to an outside emergency department complaining of headache and ascending weakness. Due to the progression of his symptoms, he was urgently transferred to our institution. Upon arrival, the patient became more lethargic and began to desaturate requiring emergency intubation. A computerized tomography (CT) scan and computerized tomography angiogram (CTA) of the head and neck were performed which did not demonstrate any acute disease. A CT scan of the cervical spine demonstrated multilevel degenerative changes without evidence of canal compromise. There was initial clinical concern that this may represent a Guillain-Barre syndrome for which the patient underwent a lumbar puncture by the neurology team demonstrating an elevated protein and mild glucose elevation. A CT scan of the cervical spine demonstrated a slight grade 1 anterolisthesis of C2 on C3 and mild loss of C6 vertebral body height. In addition, a large retro-odontoid rheumatoid pannus was noted projecting posteriorly at the craniocervical junction. Magnetic resonance (MR) of the brain was obtained revealing an irregularly shaped lesion surrounding and arising from the C1-C2 region extending cephalad and traversing the foramen magnum (Figures 1 and 2). It measured 4.1 cm in craniocaudal dimension by 2.3 cm in the anterior-posterior dimension and 1 cm in the transverse dimension. It was noted that it insinuated between the intradural segments of vertebral arteries bilaterally. This resulted in significant compression of the medulla resulting in a large cystic area in the medulla. There was marked severe stenosis at the foramen magnum and craniocervical junction with associated reactive dural enhancement. The MR of the brain demonstrated no areas of restricted diffusion nor mass lesions or abnormal enhancement. The major intracranial flow voids were maintained.

**Figure 3:**
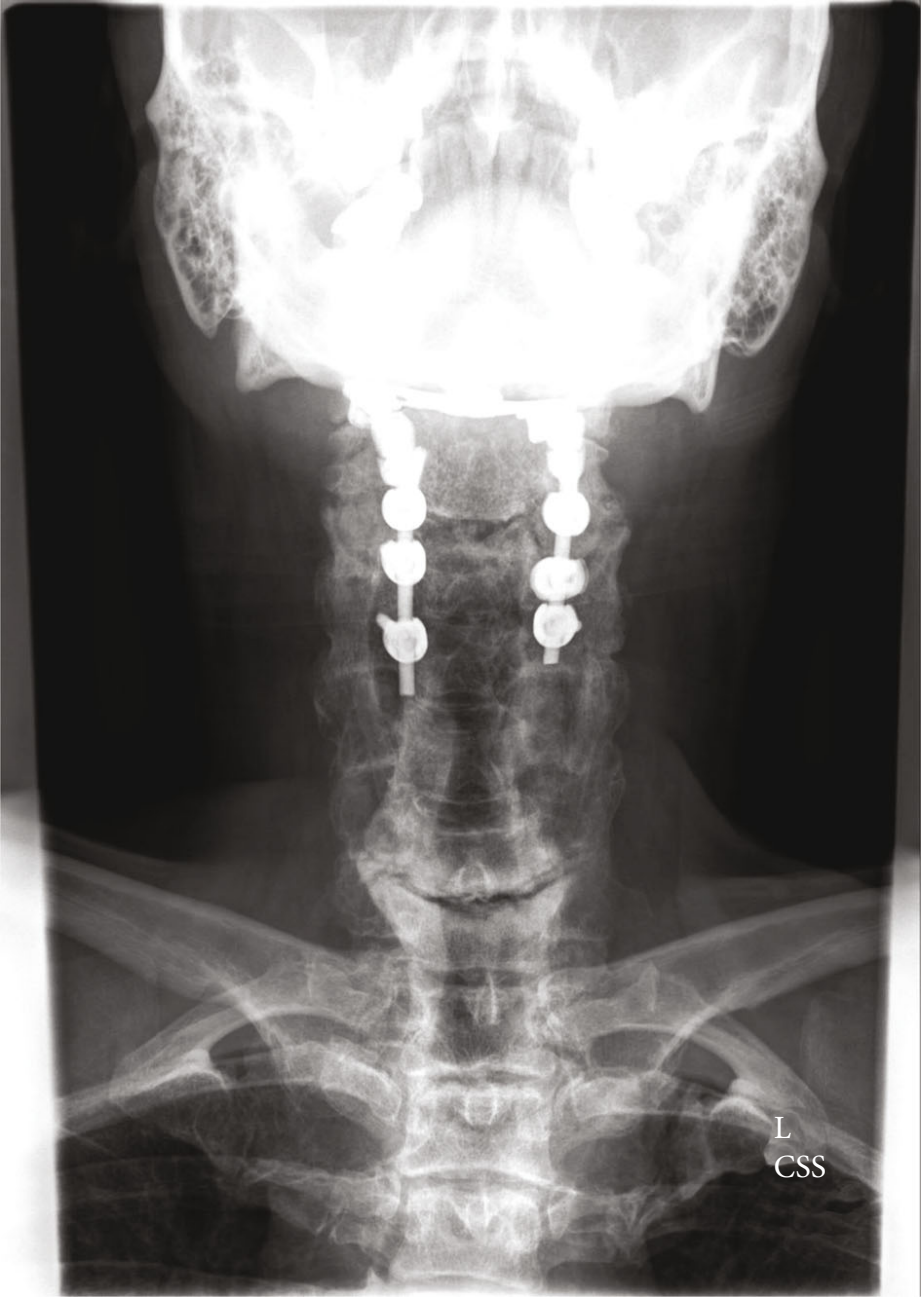
Postoperative anterior cervical spine X-ray demonstrates occipitocervical fusion with a C2-C4 lateral mass screw and posterior rod construct with the occipital plate.

**Figure 4:**
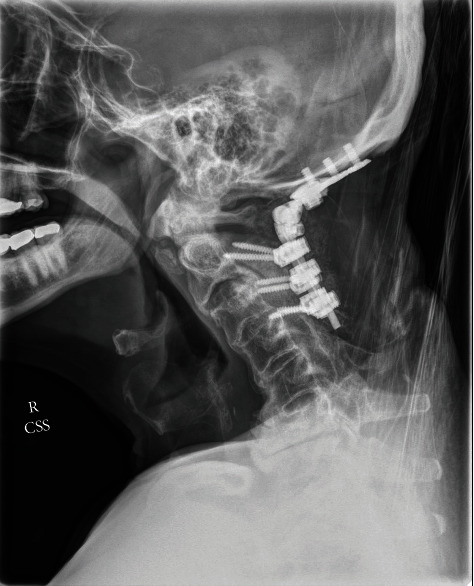
Postoperative lateral cervical spine X-ray demonstrates occipitocervical fusion with a C2-C4 lateral mass screw and posterior rod construct with the occipital plate.

**Figure 5:**
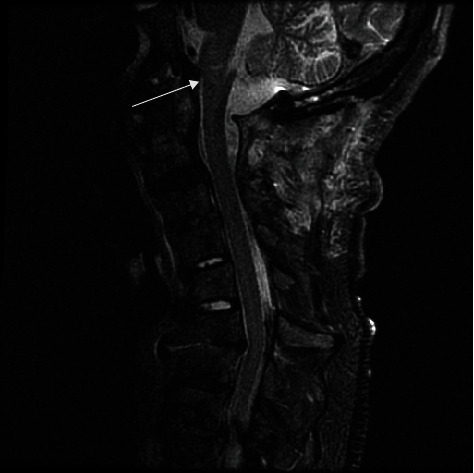
Postoperative MR cervical T2-weighted sagittal image demonstrates resolution of the cervicomedullary compression and caudal medullary cyst.

**Figure 6:**
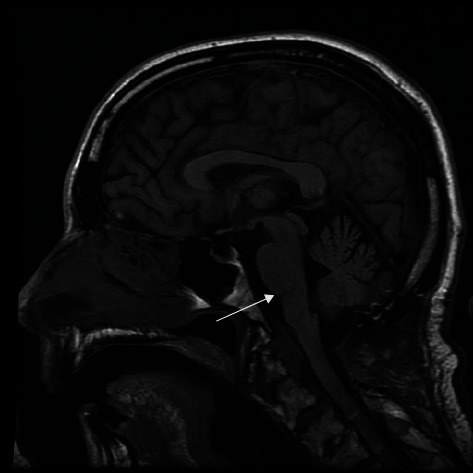
Postoperative MR brain T1 FLAIR sagittal image demonstrates resolution of the cervicomedullary compression and caudal medullary cyst.

**Figure 7:**
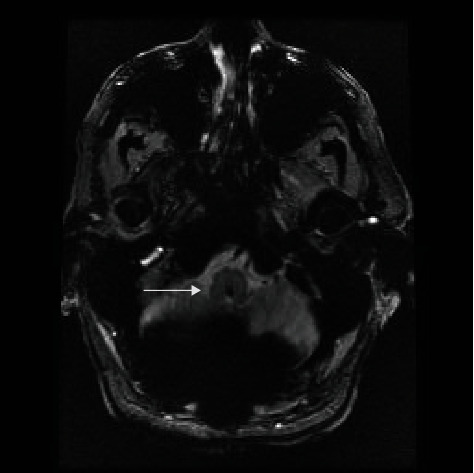
Postoperative cervical T2-weighted axial image demonstrates resolution of the caudal medullary cyst.

Given the rapid neurological progression and the imaging findings, the patient underwent an urgent suboccipital craniectomy with C1 and C2 bilateral laminectomies and occipitocervical fusion occiput to C4. Due to the acute neurological deterioration upon arrival to our institution requiring emergency intubation and the severe medullary compression, the patient was not able to safely undergo preoperative dynamic cervical imaging. The medullary compression and the listhesis of C2 on C3 prompted the surgical decision making to fuse the patient from the occiput to C4 to optimize stabilization and prevent further brainstem injury. The patient did require tracheostomy and percutaneous gastrostomy for nutrition postoperatively. The patient required inpatient rehabilitative services. At follow-up four months after surgery, the patient was ambulatory. The tracheostomy was decannulated, and the feeding tube had been removed and he was eating a normal diet orally. He did have some residual numbness in the buttocks and hands that was slowly improving. The remainder of neurologic examination was intact. A postoperative MR scan demonstrated marked reduction in the size of the pseudo-pannus at the craniocervical junction (Figures 3–5). The cystic component in the medulla resolved without any remaining deformity of the brainstem. The area of abnormal signal within the medulla resolved. Postoperative X-rays demonstrated the occipital cervical posterior construct Figures 6 and 7).

## 3. Discussion

Retro-odontoid cysts are rare clinical entities often occurring in elderly patients. Differential diagnosis includes pyogenic infections, tumor, synovial cysts, and calcium pyrophosphate deposition within the ligaments. These clinical entities can result in significant neurological morbidity and death if not aggressively treated. Lesions that arise around the odontoid resulting in significant mass effect on the brainstem and spinal cord have been associated with trauma, hemodialysis, diffuse idiopathic skeletal hyperostosis, and rheumatoid arthritis [9–18]. Retro-odontoid cysts are a separate clinical entity. Mechanisms proposed at the etiology for these lesions include chronic instability and pseudogout [1–3]. Various surgical techniques have been described including posterior laminectomy, far lateral, posterior intradural approach, and open and endoscopic transoral/transnasal approaches [1–8].

The anterior approaches have the advantage of directly accessing the pathology; however, they are reported to have higher morbidity rates particularly in the elderly patient [3, 14, 15]. Often, the anterior approach requires posterior fixation and stabilization [18]. As in our case, posterior occipitocervical fusion alone can result in reduction or elimination of the retro-odontoid cyst and decompression of the cervicomedullary junction [8]. Lateral approaches to lesion at the cervicomedullary junction have also been described but are more commonly used for neoplastic lesions [16, 17]. Madhavan et al. reported on three elderly patients with a retro-odontoid cyst approach posteriorly via the transdural approach [3]. In two of the three cases, posterior stabilization was performed. All patients did well with 1 patient requiring repair of a dorsal pseudomeningocele 3 months following surgery. Imaging demonstrated resolution of the cyst and cervical medullary compression. In our case, the cyst resolved with fusion and stabilization, which suggests that the cyst was related in part to abnormal mobility or instability. The expansion of the use of endoscopic techniques for spinal surgery pioneered greatly by Fessler et al. described the case of a transoral-transpharyngeal approach using the endoscope to resect an odontoid mass. The patient did require a posterior fusion with resultant good surgical outcome [15]. In our case, the patient underwent a posterior occipitocervical fusion without resection of the retro-odontoid cyst. His postoperative imaging demonstrated resolution of the cervicomedullary compression as well as the cyst that had developed within the medulla. The patient made a good neurologic recovery.

## 4. Conclusions

Retro-odontoid cysts are not an uncommon clinical entity. The exact etiology of these is not completely understood. Patients can develop significant neurologic morbidity due to the compression at the cervicomedullary junction. Surgical intervention is necessary to halt and hopefully reverse neurological deterioration. In this case, posterior occipitocervical fusion alone resulted in the resolution of the patient's symptoms and reversal of imaging findings.

## Data Availability

The academic data used to support the findings of this study are included within the article.
